# Invasive Predator Management Can Mitigate the Impacts of Fire and Low Rainfall for Some Herpetofauna

**DOI:** 10.1002/ece3.72755

**Published:** 2025-12-26

**Authors:** Kristina J. Macdonald, Bronwyn A. Hradsky, Tim S. Doherty, Don A. Driscoll

**Affiliations:** ^1^ School of Life and Environmental Sciences, Deakin University Melbourne Victoria Australia; ^2^ Research Institute for the Environment and Livelihoods Charles Darwin University Darwin Northern Territory Australia; ^3^ School of Agriculture, Food and Ecosystem Sciences University of Melbourne Melbourne Victoria Australia; ^4^ School of Life and Environmental Sciences The University of Sydney Sydney New South Wales Australia; ^5^ Biodiversity and Conservation Science Department of Biodiversity, Conservation and Attractions Perth Western Australia Australia

**Keywords:** amphibian, drought, lethal baiting, planned burn, reptile, threat interaction

## Abstract

Effective wildlife conservation relies on understanding how threats and associated management actions can interact to affect biodiversity. However, the impacts of concurrent disturbances on wildlife can be complex and difficult to predict. Altered fire regimes, invasive predators, and reduced rainfall are major threats to herpetofauna, but no research has investigated their interactive effects on any reptile or amphibian species to date. We undertook a natural experiment employing a control‐impact design in southern Australia to examine the combined and independent effects of prescribed fire, invasive red fox ( *Vulpes vulpes* ) management and low rainfall on reptile and amphibian richness and abundance. We found negative effects of prescribed fire on reptile species richness and the capture rates of four (out of five) herpetofauna species. Fox management had a positive effect on three skink species and a negative effect on the fourth. Our results demonstrate important interactions for some species. For example, low rainfall exacerbated the negative effects of prescribed fire for two species, while fox management mitigated the negative effects of prescribed fire and low rainfall for two species. This is the first study investigating the interactive effects of these three key threats on herpetofauna. Our discoveries demonstrate that investigating interactive effects can help explain why species have spatially and temporally variable responses to disturbances. Our results suggest that it may be beneficial to avoid prescribed fires and increase invasive predator management during climatically stressful periods.

## Graphical abstract

Fire, invasive predators, and drought are major threats for herpetofauna. These key global drivers are known to interact, yet, research investigating their interactive effects on wildlife remains limited. We found the management of invasive predators could partially mitigate the negative effects of prescribed fire and low rainfall for some species.

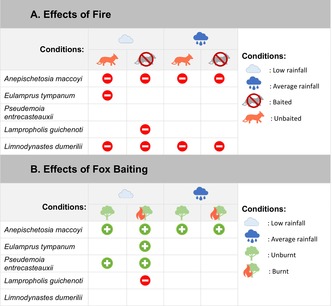

## 1 Introduction

Climate change is driving biodiversity declines by increasing the extent and intensity of anthropogenic and natural disturbances (IPBES 2019; Parmesan et al. 2022). Disturbances are also increasingly co‐occurring, which can have detrimental effects on ecosystem resilience (Didham et al. 2007, Sergio et al. 2018; Newman 2019). Understanding the effects of multiple, concurrent disturbances on wildlife is fundamental for disturbance ecology and conservation management (Driscoll et al. 2016; Côté et al. 2016; Geary et al. 2019).

Inappropriate fire regimes, invasive predators, and drought are each important disturbance types that can threaten wildlife (Clavero and García‐Berthou 2005; Doherty et al. 2016; Vicente‐Serrano et al. 2020; Kelly et al. 2020). Although fire is a natural process, changing global fire regimes are resulting in higher fire frequency, severity, and extent in many regions (Moreira et al. 2020; Nolan et al. 2020; Pausas and Keeley 2021). In addition to wildfire, prescribed fire is a key tool used by land management agencies (Boer et al. 2009) which can also contribute to changing fire regimes. Prescribed fire often aims to manage wildfire risk (Boer et al. 2009), and in some cases is used to restore ecosystems and promote biodiversity (Fair and Henke 1997). However, inappropriate fire regimes can threaten biodiversity by increasing species' extinction risk and reducing ecosystem resilience (Keeley and Pausas 2019; Kelly et al. 2020; Doherty et al. 2024).

Invasive species are also key drivers of biodiversity declines and yet, invasion pathways are increasing globally, and key management targets are not being met (IPBES 2019; Henriksen et al. 2024). Invasive species can have varied impacts on native biodiversity, including via direct mortality and through the alteration of community dynamics and habitat structure. Invasive predators are of particular concern regarding native species loss (Doherty et al. 2016), and lethal control is often used by land managers with the aim of reducing invasive predator populations and protecting native species (Russell et al. 2016). However, there can be large variability in management effectiveness and resulting biodiversity outcomes (Rendall et al. 2021; Pearson et al. 2022).

Climatic extremes, including periods of low rainfall and drought, are becoming more frequent, prolonged and severe due to climate change (Kirono et al. 2020; Parmesan et al. 2022). Drought conditions can affect wildlife habitat via impacts on vegetation health and structure (Pratt et al. 2014; Keeley et al. 2022) and drive direct mortality through desiccation (McMenamin et al. 2008; Wu et al. 2024). In addition, wildlife can face slower recovery under drought conditions, which can affect their resilience to additional concurrent and future threats (Vicente‐Serrano et al. 2020). Hence, droughts can generate widespread effects with potential for interaction with other disturbances.

Wildlife responses to multiple disturbance types are often complex and can include synergistic and non‐linear responses that are difficult to predict (Didham et al. 2007; Côté et al. 2016). Inappropriate fire regimes, invasive predators and low rainfall are increasingly co‐occurring and have the potential to interact. For example, fire‐related simplification of habitat structure can improve the ability of some invasive predators to hunt prey (Doherty et al. 2022) which may in turn exacerbate native species' declines (Hradsky 2020). Likewise, drought can alter the impact of fires, via increased vegetation flammability and fire intensity (Pausas and Keeley 2021). In turn, severe drought and high severity fire can have more substantial impacts on wildlife (Jolly et al. 2022; Driscoll et al. 2024). Drought can also influence predator–prey dynamics by altering habitat structure and prey availability (McDowell and Medlin 2009). Understanding how prescribed fire, invasive predator management and low rainfall interact to affect wildlife is fundamental for conservation management (Driscoll et al. 2016; Kelly et al. 2020; Bellard et al. 2022).

Australia is a global hotspot for fire, invasive predators, and drought (Murphy et al. 2013; Nguyen et al. 2021; Bellard et al. 2022), and its reptile and amphibian assemblages are highly diverse and endemic (Uetz et al. 2022; Frost 2023). Attempts to predict herpetofauna responses to threats have proven challenging (Driscoll and Henderson 2008; Nimmo et al. 2012; Nowakowski et al. 2017; Macdonald et al. 2023), highlighting both the complexity of herpetofauna responses to threatening processes and the major challenges for effective herpetofauna conservation (Geary et al. 2019). A recent review on Australia's threatened reptiles called for urgent research into how fire interacts with additional stressors, including invasive predators and climate extremes (Santos et al. 2022). The invasive red fox ( *Vulpes vulpes* ; hereafter ‘fox’) has had severe impacts on Australian biodiversity and preys on at least 20% of Australia's threatened terrestrial reptile species (Woinarski et al. 2015; Stobo‐Wilson et al. 2021). Further, prescribed fire is a common management tool in Australia, aimed at mitigating the risk of wildfires (Gazzard, Walshe, et al. 2020). However, the combined effects of prescribed fire and additional stressors on wildlife remain poorly studied (Driscoll et al. 2010; Santos et al. 2022). Fox activity can increase in recently burnt landscapes (Hradsky 2020; Doherty et al. 2023), which may exacerbate the impacts of foxes on some wildlife by removing shelter for prey species, thereby increasing fox hunting efficiency. However, research on fox‐fire interactions thus far has primarily focused on native mammalian prey responses (e.g., Hradsky et al. 2017; Geary, Tulloch, et al. 2023; Menon et al. 2025). The interactive effects of fire, invasive predators (or predator management), and climatic stressors remain unstudied for any reptile or amphibian species.

We conducted a repeated natural experiment to examine the effects of prescribed fire, fox management and low rainfall on the richness and abundance of herpetofauna in southern Australia. Studying prescribed fire gave us the opportunity to examine multiple fires within a single landscape in areas with and without fox management. The study was conducted during a year with below average rainfall and was repeated 2 years later during a year of average rainfall. We expected that prescribed fire would negatively impact the abundance and richness of reptiles and amphibians in the short‐term, and that these impacts would be greater within areas subjected to higher fire severity, and under low rainfall conditions. In addition, we hypothesised that the lethal baiting of foxes would have a positive effect on the abundance and richness of herpetofauna, particularly at burnt sites.

## 2 Methods

### 2.1 Study Region

We conducted this project in the Glenelg Region of southwest Victoria, Australia (−38.10, 141.54). This region has mean minimum and maximum annual temperatures of 9° and 18°C, respectively, and a mean annual rainfall of 840 mm (Bureau of Meteorology 2023).

The Victorian State Government has undertaken prescribed burning in Glenelg since the 1970s (Gazzard, McLeod, et al. 2020). The current annual prescribed fire program in this region aims to reduce the risk of wildfire in summer (Gazzard, McLeod, et al. 2020). Concurrently, the ongoing Glenelg Ark fox management program operates across 90,000 ha of native forest reserves. The southern half of this area is subjected to lethal fox baiting every second week using poison‐baits containing 3 mg of sodium fluoroacetate (‘FoxOff’, Animal Control Technologies, Somerton) buried at 1‐km intervals along accessible forest tracks and roads. Rees et al. (2023) found that fox occupancy was 27%–70% lower in the baited landscape compared to the unbaited landscape within this program area.

Glenelg contains large areas of natural forest within a matrix of cleared pastoral land and native and exotic timber plantations. Our study was conducted in protected native *Eucalyptus* forests from two similar ecological vegetation classes: lowland forest (dominated by *E. willisii*, *E. obliqua* and *E. baxteri* ) and herb‐rich foothill forest (dominated by *E. ovata* and *E. viminalis* ). These vegetation classes share similar understories that include *Xanthorrhoea minor, Microlaena stipoides, Lomandra sororia, Pteridium esculentum* and *Senecio tenuiflorus* (Department of Sustainability and Environment 2004).

### 2.2 Study Design

**FIGURE 1:**
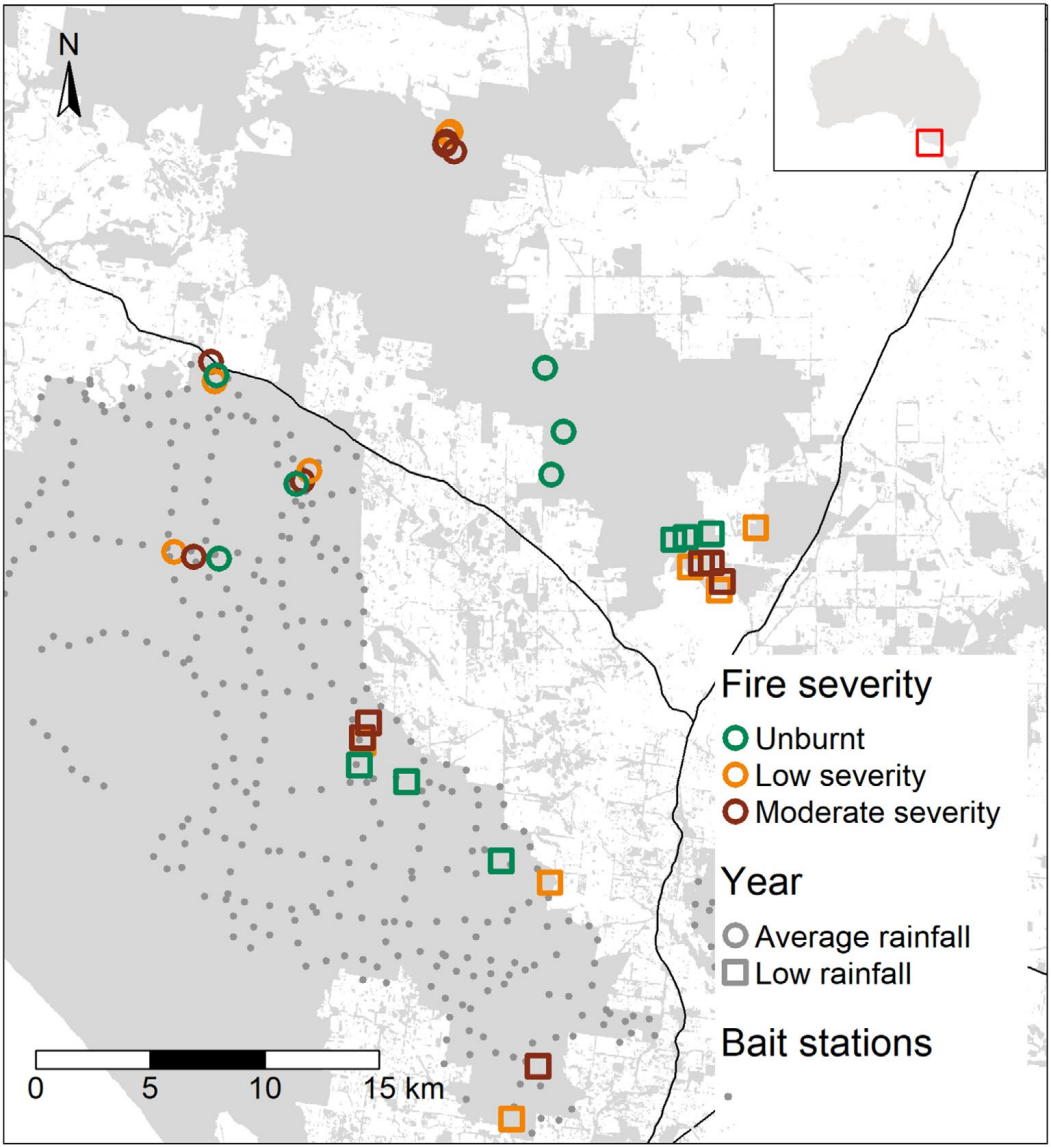
The locations of field sites. The map depicts native vegetation with grey polygons and main roads with black lines. The grey points show lethal fox baiting stations. Field sites are denoted with symbols. Fire levels: Moderate severity (red), low severity (orange) and long unburnt (blue), and season: Average rainfall (circle), low rainfall (square).

We used a replicated, crossed control‐impact design of prescribed fire (hereafter fire) and lethal fox baiting (hereafter baiting). We established sites across nine independent prescribed fires, which each burnt 100–800 ha. We used government fire severity maps to identify potential sites of contrasting fire severity. The burnt areas typically comprised predominately low canopy scorch (< 20%), with smaller areas of medium (20%–80%) and high (> 80%) canopy scorch. During site selection, we calibrated the fire severity of sites on ground by assessing the understory vegetation within the site footprint (0.45 ha). We classified sites as ‘moderate severity’ when the entire understory had been burnt (i.e., no live foliage on shrubs), or ‘low severity’ when the fire was patchier, and parts of the understory remained unburnt. Unburnt sites were established in areas that had remained unburnt for at least 30 years and were spatially clustered near burnt sites where possible (Figure 1).

Prescribed fire was undertaken in autumn, and we established our sites 7–9 months post‐fire to align with higher reptile activity (i.e., summer). Sites were established in areas with baiting (‘baited’; *n* = 18) and without baiting (‘unbaited’; *n* = 17) that were subjected to one of the three fire levels: unburnt (at least 30 years since fire), low severity, and moderate severity (Figure 1). We collected data in two austral summer seasons (2019/20 and 2021/22) to maximise the number and spatial spread of sites (Figure 1). The first season (‘low rainfall’) coincided with the end of a severe drought period (Nguyen et al. 2021) when local annual rainfall was 18% below average (685 mm), while the second season (‘average rainfall’) was conducted approximately 2 years post‐drought, during an average rainfall year (844 mm; Bureau of Meteorology 2023). We were unable to conduct surveys during the 2020/21 season due to COVID restrictions.

Rainfall and year were synonymous in our experimental design, and sites within the low rainfall year were located further south within our study area due to prescribed fire locations (Figure 1). Therefore, we could not rule out other unmeasured differences between the 2 years contributing to observed effects, although rainfall was the most prominent difference. We were able to control for spatial variation in the statistical analysis (see ‘Statistical Analysis’) and sites were otherwise comparable, containing the same vegetation types and similar elevation (mean elevation of 70 m and 130 m across low and average rainfall sites respectively).

On average, sites were 960 m (range: 100–2500 m) from the next closest site. At each site, we installed 15 aluminium flywire drift fences (10 m long) in a 5 × 3 grid covering 0.45 ha. At the centre of 10 fences there was one pitfall trap (20 L bucket), while there was a pair of funnel traps (one either side of the fence) at the remaining five fences. In total, 10 pitfall traps and 10 funnel traps were installed at each site, with funnel traps installed at each corner and the middle fence.

### 2.3 Survey Methods

In the first season (2019/20) we surveyed 18 sites in January (11th– 22nd), and again in February (4th–16th) and March (3rd– 15th). In the second season (2021/2022) we surveyed 17 different sites in December (3rd–15th), and again in January (19th–30th) and February (15th–27th). For logistical reasons, we divided sites into two survey groups per season (split evenly across treatments). Each survey group was intended to be surveyed for five consecutive trap nights per survey period, with traps checked twice per day. However, sites were closed during forecast heavy rain, wind, or storm warnings. This resulted in variable sampling effort (2–5 trap nights per survey). All captured vertebrate species were recorded, and all lizards and amphibians (weighing > 1 g) were tagged with visible elastomer. Recaptured animals made up a low proportion of captures (2.5%).

### 2.4 Statistical Analysis

We fitted generalised linear mixed effects models to examine the effects of prescribed fire, baiting and rainfall on reptile naïve species richness (hereafter: species richness), amphibian species richness, the capture rates of lizards, the capture rates of amphibians, and the capture rates of each species with more than 50 captures. The latter comprising four reptile species: *Lampropholis guichenoti* (pale‐flecked garden skink), *Eulamprus tympanum* (southern water skink), *Anepischetosia maccoyi* (Maccoy's skink) and *Pseudemoia entrecasteauxii* (southern grass skink), and one frog species: *Limnodynastes dumerilii* (pobblebonk frog) (Table S1). All response variables were calculated for each monthly trapping session, and we used a Poisson distribution for all models except for reptile species richness where we used a generalised Poisson distribution which improved model diagnostics (simulated QQ plot residuals).

We had two alternative global models which included different fire predictor variables: ‘fire status’ to test whether herpetofauna showed a consistent response to fire (regardless of severity), and ‘fire severity’ to test whether responses differed between low and moderate severity fire (Table 1). Our global models both included the three‐way interaction between the fire predictor, baiting and rainfall (Table 1). In addition to our disturbance predictors, we expected that survey temperatures could influence capture rates (Spence‐Bailey et al. 2010). We accounted for this in our models with a main effect of the mean maximum daily temperature over the 5 days of each survey period, excluding any days when traps were closed (Bureau of Meteorology 2023).

**TABLE 1:** Descriptions of the fixed effect model structures for each sub‐global generalised linear mixed model used to fit response variables.

Global Model 1	Fire status (unburnt and burnt) × baiting (unbaited and baited) × rainfall (low rainfall and average rainfall) + temperature (continuous)
Global Model 2	Fire severity (unburnt, low severity, and moderate severity) × baiting (unbaited and baited) × rainfall (low rainfall and average rainfall) + temperature (continuous)

In all models we included three random intercept terms: ‘site’, ‘survey period’ and ‘site cluster’. Site (35 levels) accounted for the three repeated surveys undertaken at each site. Survey period (12 levels comprising the two survey periods in each of the three sampling months per year) was used to account for additional possible detection variability due to differences in survey conditions across survey periods. ‘Site cluster’ (10 levels containing 2–7 sites) was used to account for similarities among spatially clustered sites and variation across the landscape. Site clusters consisted of sites in close spatial proximity containing a single fire block and environmental vegetation class (Figure S1). As an exception, one cluster contained three unburnt sites from both vegetation classes.

For each model assessing capture rates, we included an offset term for the number of survey nights to account for survey effort variation. We did not use an offset term in our species richness models because the number of species does not proportionally increase with survey effort.

For each response metric, we first assessed the alternative global models using Akaike Information Criterion corrected for small sample size (AICc). For one species, *Anepischetosia maccoyi*, Global Model 2 did not converge and for all other response metrics, Global Model 1 was better supported with a delta AICs value greater than two (Table S2) (Burnham and Anderson 2004). Therefore, we used Global Model 1 as the global model for each response metric. Next, we undertook likelihood ratio tests to assess whether the global model had more support compared to the null model (model without any fixed effect terms). Similar to Muff et al. (2022), we consider evidence of support as weak (*p* value 0.10–0.05), moderate (*p* value 0.05–0.01), or strong (*p* value < 0.01).

We undertook fixed effects contrasts for each supported global model to assess the overall influence of the fixed effects. When there was at least weak support for an interaction effect, we undertook pairwise comparisons to assess the differences between the levels of fixed effects using the ‘sidak’ method to adjust *p*‐values; this approach reduces the probability of making a Type 1 error.

We used RStudio v.2022.7.2.576 and R v.4.2.1. (R Core Team 2022) for analysis. We used glmmTMB package v.1.1.7 (Brooks et al. 2017) to fit models, DHARMa package v.0.4.6 (Hartig 2022) for model fit and diagnostics and lrtest package v.0.9–40 (Zeileis and Hothorn 2002) to undertake Likelihood Ratio Tests. We undertook fixed effect contrasts and pairwise comparisons using emmeans package v.1.8.2 (Lenth 2022) and used ggplot2 package v.3.4.1 (Wickham 2016) and tmap package v.3.3–3 (Tennekes 2018) to create figures.

## 3 Results

Across the 35 sites and 9240 trap nights, we captured 1209 unique individuals from eleven reptile and eight amphibian species (Table S1).

### 3.1 Species Richness & Total Capture Rate Responses

**FIGURE 2:**
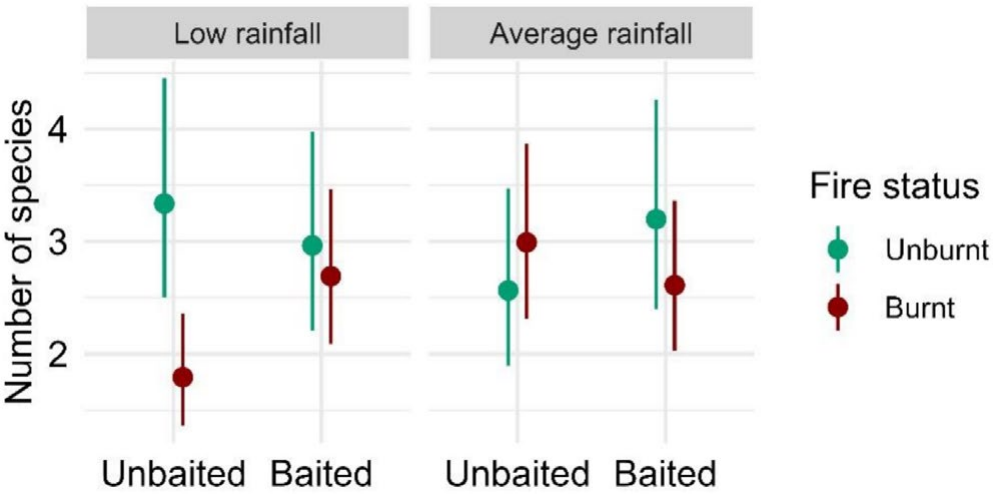
Generalised linear mixed model results showing the estimated mean richness of reptiles (and 95% confidence intervals) against the three‐way interaction of fire status, baiting and rainfall.

We found strong support and evidence for a three‐way interaction between fire status, baiting and rainfall on reptile species richness (*F* = 9.05, *p* < 0.001, Tables S3 and S4). At burnt sites without fox baiting, mean reptile species richness was 30%–46% lower under low rainfall conditions compared to other treatment combinations (Figure 2). This result was contributed to by a negative effect of fire within unbaited sites under low rainfall conditions (1.86 ± 0.28, *p* < 0.001) and a positive effect of baiting within burnt sites under low rainfall conditions (0.67 ± 0.09, *p* = 0.019; Table S5). In addition, we found weak evidence for a positive effect of rainfall on reptile richness within burnt, unbaited sites (0.60 ± 0.12, *p* = 0.098; Table S5).

For total lizard capture rates, the global model did not perform better than the null model (Table S3). Likewise, the global model did not perform better than the null model for amphibian species richness or total amphibian capture rates (Table S3).

### 3.2 Species Responses

**FIGURE 3:**
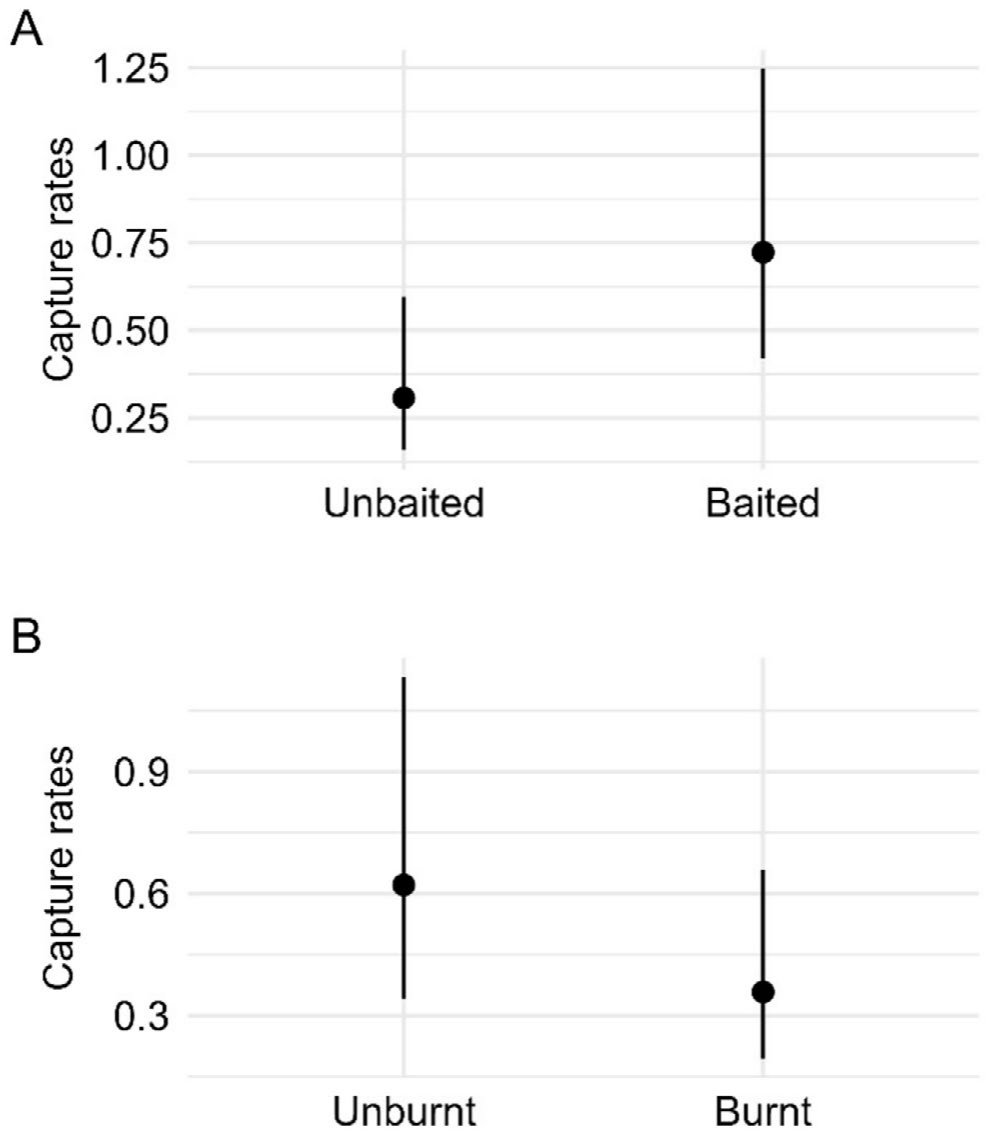
Generalised linear mixed model results showing the estimated mean capture rates of *Anepischetosia maccoyi* (Maccoy's skink) (and 95% confidence intervals) against (A) baiting (with results averaged over fire status levels: Unburnt, burnt) and (B) fire status (with results averaged baiting levels: Baited, unbaited).

The global model was better supported than the null model for all species (Table S3). We found a main effect of baiting and fire on the capture rates of *Anepischetosia maccoyi* (Maccoy's skink) (Tables S4 and S5). We found strong evidence for a positive effect of fox baiting on the capture rates of *A. maccoyi* (*F* = 7.25, *p* = 0.007, Table S4), with mean capture rates 57% lower within unbaited sites (Figure 3a). In addition, we found weak evidence for a negative effect of fire on the capture rates of *A. maccoyi* (*F* = 3.06, *p* = 0.08, Table S4), with mean capture rates 42% lower within burnt sites (Figure 3b).

**FIGURE 4:**
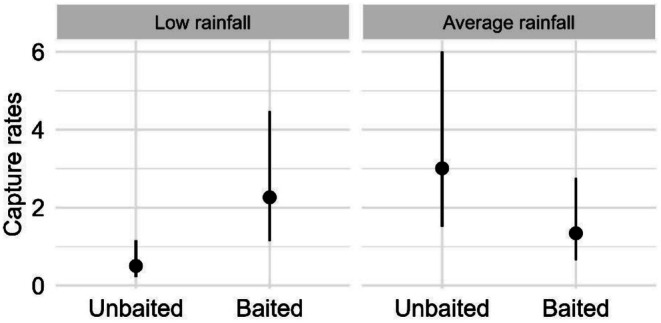
Generalised linear mixed model results showing the estimated mean capture rates of *Pseudemoia entrecasteauxii (*southern grass skink) (and 95% confidence intervals) against the two‐way interaction between baiting and rainfall.

We found strong evidence for a two‐way interaction between baiting and rainfall on the capture rates of *Pseudemoia entrecasteauxii* (southern grass skink) (*F* = 10.50, *p* = 0.001; Table S4). Mean capture rates of *P. entrecasteauxii* were lowest within unbaited sites during low rainfall conditions (Figure 4). We found strong evidence for a positive effect of baiting within the low rainfall year (0.22 ± 0.12. *p* = 0.016; Table S5), whereby mean capture rates were 77% lower within unbaited sites, compared to baited sites. In addition, we found a positive effect of rainfall, whereby mean capture rates were 83% lower within unbaited sites under low rainfall conditions compared unbaited sites within average rainfall conditions (0.17 ± 0.09, *p* = 0.005; Table S5).

**FIGURE 5:**
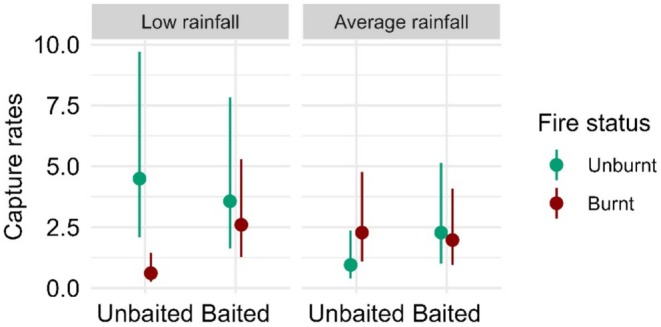
Generalised linear mixed model results showing the estimated mean capture rates of *Eulamprus tympanum (*southern water skink) (and 95% confidence intervals) against the three‐way interaction of fire status, baiting and rainfall.

For *Eulamprus tympanum* (southern water skink), we found strong evidence of a three‐way interaction between fire status, baiting and rainfall (*F* = 13.20, *p* < 0.001, Table S4). The mean capture rates of *E. tympanum* were lowest under low rainfall conditions, within sites that were both burnt and unbaited, whereby mean capture rates were 36%–86% lower compared to other treatment conditions (Figure 5). We found strong evidence for a positive effect of baiting within burnt sites under low rainfall conditions (0.24 ± 0.09, *p* = 0.001; Table S5). In addition, we found strong evidence for a negative effect of fire within unbaited sites under low rainfall conditions (7.36 ± 2.93, *p* < 0.001; Table S5).

**FIGURE 6:**
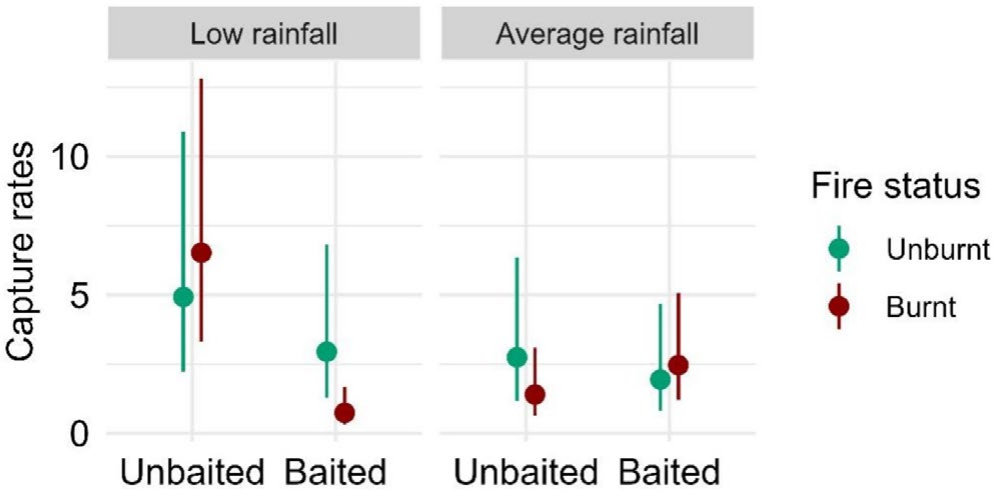
Generalised linear mixed model results showing the estimated mean capture rates of *Lampropholis guichenoti* (pale‐flecked garden skink) (and 95% confidence intervals) against the three‐way interaction of fire status, baiting, and rainfall.

For *Lampropholis guichenoti* (pale‐flecked garden skink), we found strong evidence for a three‐way interaction between baiting, fire status and rainfall in the top ranked model (*F* = 9.68, *p* = 0.002; Table S4), whereby mean capture rates were 1.3–8.8 times greater under low rainfall conditions within burnt‐unbaited sites (Figure 6). These results were contributed to by a negative effect of baiting on *L*. *guichenoti* capture rates within burnt sites under low rainfall conditions (8.83 ± 3.25, *p* = < 0.001; Table S5), a negative effect of fire within baited sites under low rainfall conditions (3.99 ± 1.77, *p* = 0.022; Table S5), and a negative effect of rainfall within burnt‐unbaited sites (4.66 ± 2.50, *p* = 0.049; Table S5).

**FIGURE 7:**
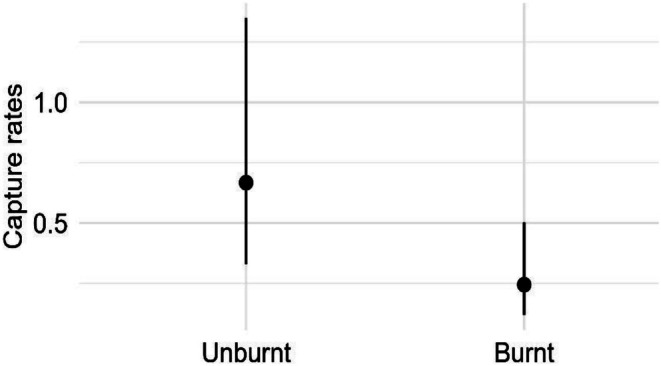
Generalised linear mixed model results showing the estimated mean capture rates of *Limnodynastes dumerilii* (pobblebonk) (and 95% confidence intervals) against fire status.

For *Limnodynastes dumerilii* (pobblebonk frog), we found strong evidence for a negative main effect of fire on capture rates (*F* = 9.16, *P* = 0.002; Table S4), whereby mean capture rates were 63% lower within burnt sites compared to unburnt sites (Figure 7).

## 4 Discussion

Our study is the first to assess the concurrent impacts of fire, invasive predator management and rainfall on reptiles and amphibians. Prescribed fire had a negative effect on reptile species richness (Figure 2) and the capture rates of four species (Table 2). However, for some species the negative effects of fire were only apparent under low rainfall conditions (Table 2). Likewise, the effects of fox baiting on species' capture rates were most observed in conjunction with low rainfall and fire (Table 2).

**TABLE 2:**
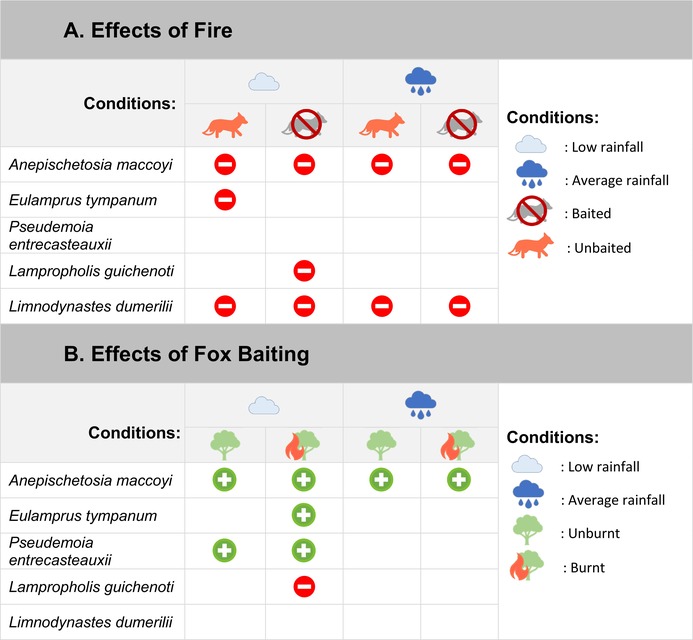
Summarised results of the effects of (A) fire status and (B) fox baiting on species' capture rates across additional disturbance conditions.

*Note:* 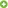 Significant positive effect of (A) fire status or (B) fox baiting on mean capture rates. 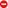 Significant negative effect of (A) fire status or (B) fox baiting on mean capture rates.

Our results suggest that low rainfall can exacerbate the effects of prescribed fire and invasive predators on some reptiles in our study system. Climatic stressors such as drought can exacerbate the effects of fire (Driscoll et al. 2024) and slow wildlife recovery (Vicente‐Serrano et al. 2020). Low rainfall can also have indirect effects on animals, including via changes in predator–prey dynamics (McDowell and Medlin 2009). For example, Catling (1988) found that foxes consumed more reptiles under drought conditions and suggested that this was due to lowered abundance of rabbits, which are preferred prey of foxes. Therefore, greater fox activity in burnt areas (Hradsky 2020) and prey switching under reduced rainfall conditions could both lead to higher predation pressure.

Other factors may have also contributed to the observed differences between the low and average rainfall years. As a natural experiment, the locations of prescribed fires were designated by land managers, limiting our ability to control for spatial and temporal variables in the field design. We were able to control for spatial clustering within our statistical analysis, accounting for some of the spatial confounding (see ‘Methods’ for details). Furthermore, the breaking of the 2019 drought was the largest climatic difference between our two seasons and no other influential temporal changes were identified. Note that we used a different set of sites in the second year to increase the number of statistically independent sites. If we had surveyed the same set of sites in the second year, we would have had half the sample size and rainfall would have been confounded with time since fire (Year 1: low rainfall year and ~1 year since fire, Year 2: average rainfall and ~2 years since fire). Nonetheless, further research on threat interactions across the boundary of future wet‐dry periods is warranted.

### 4.1 Species Traits

Ecological traits can play an important role in a species' ability to respond to disturbance (Keinath et al. 2017; Lazzari et al. 2022). The reproductive mode of a species may influence its resource and behavioural requirements (Blackburn 1999), which may explain why our viviparous study skinks, *E. tympanum* and *P. entrecasteauxii*, had the lowest capture rates under more disturbed conditions (Figures 4 and 5). Compared to egg‐laying species, viviparous species may be more sensitive to changes in predation pressure due to greater basking and foraging requirements, lower reproduction rates, and slower population recovery rates (Robert and Thompson 2007; Meiri et al. 2011; Lazzari et al. 2022).

Habitat specialisation can also influence how resilient species are to disturbance (Büchi and Vuilleumier 2016). Habitat generalists may be less sensitive to disturbance (Keinath et al. 2017), and thus more competitive in post‐disturbance environments. *Lampropholis guichenoti* is a widespread species and the only study species that occupies highly altered habitats (Prosser et al. 2006; Cogger 2018). Additionally, this species may be more drought tolerant compared to other sympatric species (Lunney et al. 1991). This could help explain why *L*. *guichenoti* capture rates were greater under more disturbed conditions (unbaited, burnt and under low rainfall conditions), an opposite trend to that of the other study species (Table 2, Figure 6).

In contrast to our predictions and previous research (e.g., Mac Nally et al. 2014; Evans et al. 2020), we found no effect of low rainfall on amphibian species richness or *L. dumerilii* abundance. *Limonodynastes dumerilii* is a large, burrowing frog species which may be more resilient to drying compared to smaller species (Thurman and Garcia 2019). The severity of drought could influence amphibian responses; research has documented significant population declines among amphibian species under severe drought conditions (e.g., Cayuela et al. 2016; Beranek et al. 2022), whereas the effects of drought in our study region were less severe.

### 4.2 Species Interactions

We found positive effects of fox baiting on the capture rates of three skink species, and a negative effect on the fourth (Table 2). Fox management may directly affect prey species through lowered fox predation, or via indirect pathways such as changes in species interactions including meso‐predator and competitor release (Ruscoe et al. 2011; Pearson et al. 2022). The meso‐predator release of feral cats ( *Felis catus* ) is of particular concern in the context of fox management (Geary, Wayne, et al. 2023; Rees et al. 2023). Although the Glenelg Ark fox control program reduces fox occupancy, feral cat densities are higher in some baited areas (Rees et al. 2023). In addition, lower fox activity within the Glenelg region has been associated with greater feral cat diurnal activity (Rees et al. 2024). All our study species are within the prey size range of both cats and foxes (Stobo‐Wilson et al. 2021), but it remains unstudied how meso‐predator release of cats might differentially affect sympatric prey species in fox‐baited landscapes.

Changes in the abundance and activity of native predators and competitors may also affect species' responses to fire and fox management. For Example, *L*. *Guichenoti* is preyed upon by other reptiles, including *E*. *Tympanum* (pers. observation, KJ Macdonald). Therefore, greater fox activity in burnt landscapes may lower the abundance of *E. Tympanum* and allow *L*. *Guichenoti* to increase. Comparatively little is known about fox impacts on herpetofauna compared to mammals. Although some studies have previously looked at changes in total reptile abundance in response to fox management (e.g., Hu et al. 2019), there has been little research investigating its impact on individual small lizard species. Understanding the mechanisms driving species responses to fox baiting is important for conservation management. Future research should undertake targeted dietary studies (e.g., Stomach content analysis or genetic analysis) to improve our understanding of invasive predator impacts on herpetofauna species.

### 4.3 Management Implications and Future Research

Our findings that herpetofauna capture rates can be influenced by prescribed fire, fox management, low rainfall and their interactive effects emphasise the importance of considering disturbance interactions for effective ecosystem management and biodiversity conservation (Geary et al. 2019). Lethal management of invasive species is often contentious because there can be large variability in its effectiveness and the resulting benefits for biodiversity (Rendall et al. 2021; Pearson et al. 2022). We found that fox management had a positive overall effect on one species (*A. maccoyi*) and could partially mitigate the impacts of prescribed fire or low rainfall for reptile richness and the capture rates of two of the four reptile species. Intriguingly, the fourth reptile species, the more common habitat generalist, *L*. *guichenoti*, fared worse after fire in baited than unbaited sites under the same conditions. Nevertheless, managing invasive predators may be beneficial for some native species and improve resilience during periods of additional stress, such as low rainfall conditions and after prescribed fire.

High severity fires often have greater negative effects on herpetofauna (Santos et al. 2022; Beranek et al. 2023). Surprisingly, the global model including fire severity was less supported for all response metrics. Prescribed fires are typically undertaken at lower severity with the aim to reduce negative ecological outcomes (Gazzard, McLeod, et al. 2020) and as such, we were only able to compare low and moderate severity fire. Regardless, our results highlight that prescribed fire can negatively impact species richness and abundance. We suggest that it would be beneficial to avoid prescribed fire during environmentally stressful conditions such as drought, especially in places without effective fox management.

To further our knowledge on threat interactions and their management, we suggest that future research should aim to: (1) increase spatial and temporal replication within rainfall and drought research to tease out potential confounding variables; (2) incorporate predator–prey dynamics and competition into disturbance research because many effects of disturbance may be indirect; and (3) assess individual and demographic‐level responses (e.g., reproduction, body condition, behaviour) and functional response groups. Such research is essential for understanding underlying mechanisms, improving predictive capacity, and ultimately improving conservation outcomes for herpetofauna.

## Author Contributions

**Kristina J. Macdonald:** data curation (lead), formal analysis (lead), funding acquisition (supporting), investigation (equal), methodology (equal), project administration (lead), visualization (lead), writing – original draft (lead), writing – review and editing (lead). **Bronwyn A. Hradsky:** conceptualization (equal), funding acquisition (equal), methodology (supporting), supervision (supporting), writing – review and editing (equal). **Tim S. Doherty:** methodology (supporting), supervision (equal), writing – review and editing (equal). **Don A. Driscoll:** conceptualization (equal), funding acquisition (equal), methodology (supporting), supervision (lead), writing – review and editing (equal).

## Funding

This work was supported by Parks Victoria. Australian Research Council (DE200100157, LP170101134). Holsworth Wildlife Research Endowment.

## Conflicts of Interest

The authors declare no conflicts of interest.

## Supporting information


**Data S1:** Supporting Information.

## Data Availability

Supporting data and code for this manuscript has been uploaded to Dryad: https://doi.org/10.5061/dryad.x69p8czrs.
