# Supplementary material for: Invasive Predator Management Can Mitigate the Impacts of Fire and Low Rainfall for Some Herpetofauna
Source: Ecol Evol. 2025 Dec 26;15(12):e72755. doi: 10.1002/ece3.72755 (PMC12742462; doi:10.1002/ece3.72755)
Supplement: Supplementary file 1 — Data S1: Supporting Information. [file ECE3-15-e72755-s001.docx]

**Appendix 1**

| **TABLE S1:** List of species captured and number of individuals | | |
| --- | --- | --- |
| **Scientific name** | **Common name** | **Number of individuals** |
| *Acritoscincus duperreyi* | Eastern three-lined skink | 1 |
| *Anepischetosia maccoyi* | McCoy's Skink | *75* |
| *Austrelaps superbus* | Lowland Copperhead | *2* |
| *Cercartetus nanus* | Eastern pygmy possum | *8* |
| *Crinia* spp. | Crinia species | *50* |
| *Drysdalia coronoides* | White-lipped Snake | *5* |
| *Eulamprus tympanum* | Southern Water Skink | *285* |
| *Geocrinia laevis* | Smooth froglet | *38* |
| *Lampropholis guichenoti* | Pale-flecked Garden Sunskink | *392* |
| *Limnodynastes dumerilii* | Pobblebonk frog | *69* |
| *Limnodynastes peronii* | Striped marsh frog | *2* |
| *Limnodynastes tasmaniensis* | Spotted Marsh Frog | *3* |
| *Liopholis whitii* | White’s skink | *1* |
| *Lissolepis coventryi* | Swamp skink | *1* |
| *Litoria ewingii* | Southern Brown Tree Frog | *36* |
| *Mus musculus* | House mouse | *10* |
| *Neobatrachus sudellae* | Common Spadefoot Toad | *1* |
| *Notechis scutatus* | Tiger snake | *4* |
| *Pseudemoia entrecasteauxii* | Southern Grass Skink | *255* |
| *Pseudophryne bibronii* | Bibron’s toadlet | *1* |
| *Sminthopsis spp* | Dunnart | *13* |
| *Tiliqua nigrolutea* | Blotched Blue Tongue | *2* |

| **TABLE S2:** Akaike Information Criterion values corrected for small sample size (AICc) for the two alternative global models for each response metric:  Global Model 1: fire status (unburnt and burnt) × baiting (unbaited and baited) × rainfall (low rainfall and average rainfall) + temperature (continuous). Global Model 2: fire severity (unburnt, low severity, moderate severity) × baiting (unbaited and baited) × rainfall (low rainfall and average rainfall) + temperature (continuous). | | | |
| --- | --- | --- | --- |
| **Response variable** | **Global Model 1** | **Global Model 2** | **Delta AICc** |
| Reptile richness | 333.7278 | 340.1276 | 6.3998 |
| Total lizard abundance | 655.4831 | 664.9605 | 9.4774 |
| *Anepischetosia maccoyi* | 244.6904 | did not converge |  |
| *Pseudemoia entrecasteauxii* | 397.5172 | 402.1345 | 4.6173 |
| *Eulamprus tympanum* | 429.5211 | 438.1501 | 8.629 |
| *Lampropholis guichenoti* | 492.2535 | 501.967 | 9.7135 |
| Amphibian richness | 247.8236 | 255.3156 | 7.492 |
| Total amphibian abundance | 414.2268 | 421.0993 | 6.8725 |
| *Limnodynastes dumerilii* | 245.4051 | 251.6874 | 6.2823 |

**FIGURE S1:** The locations of site ‘clusters’: black circles. Clusters consisted of sites in close spatial proximity containing a single fire block and environmental vegetation class. As an exception, one cluster contained three unburnt sites from both vegetation classes. The map depicts native vegetation with grey polygons and main roads with black lines. The grey points show lethal fox baiting stations. Field sites are denoted with symbols. Fire levels: moderate severity (red), low severity (orange) and long unburnt (blue), and season: average rainfall (circle), low rainfall (square).


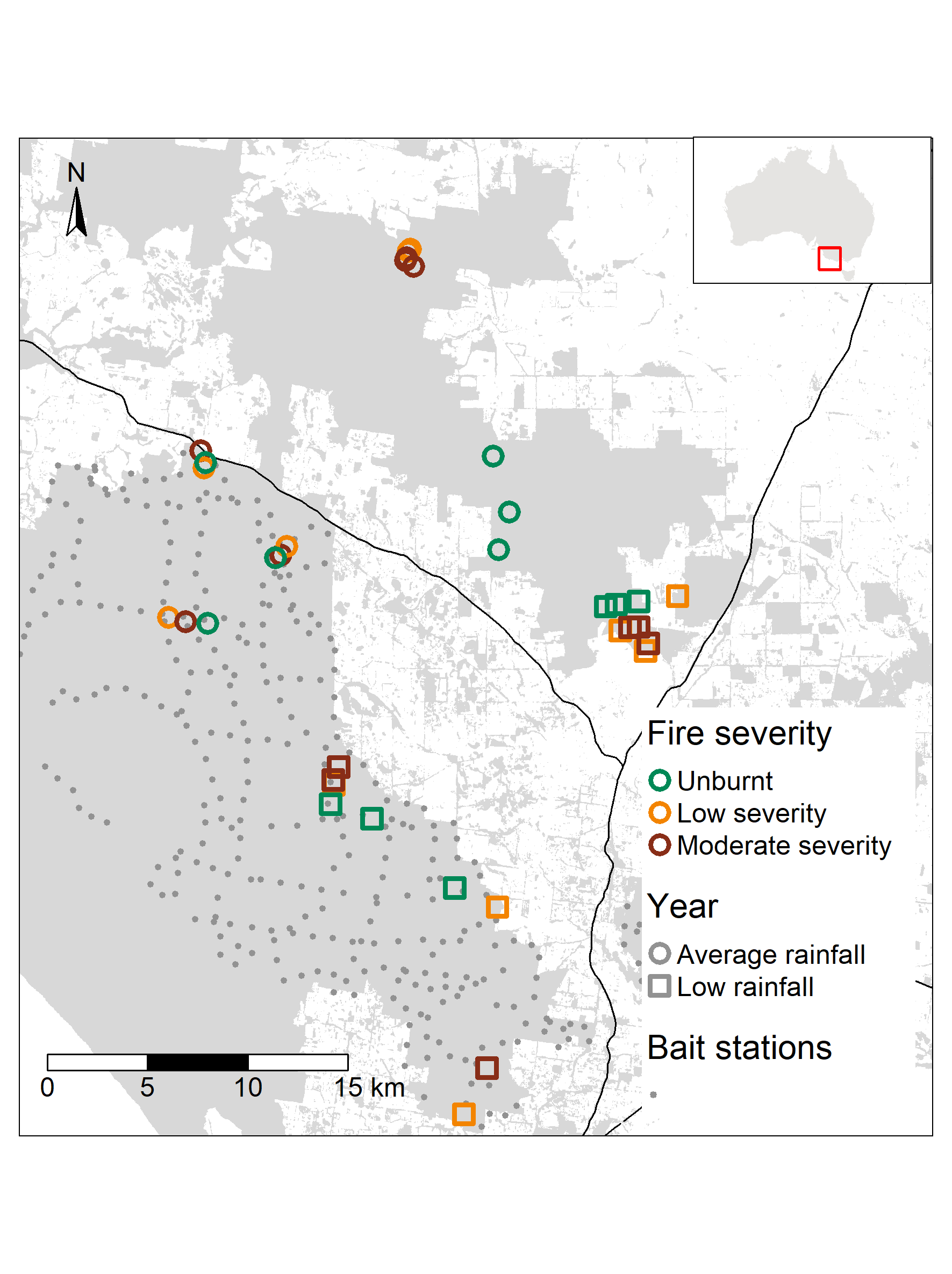


| **Table S3:** Results from the likelihood ratio tests comparing the null model and global model for each response metric. | | | |
| --- | --- | --- | --- |
| **Response variable** | **LogLik: Null** | **LogLik: Global** | **P value** |
| Reptile richness | -161.29 | -151.86 | **0.01571** |
| Total lizard abundance | -316.58 | -314.05 | 0.7511 |
| Anepischetosia.maccoyi | -117.25 | -108.65 | **0.02817** |
| Pseudemoia.entrecasteauxii | -192.38 | -185.06 | **0.0667** |
| Eulamprus.tympanum | -215.3 | -201.06 | **0.0003929** |
| Lampropholis.guichenoti | -244.55 | -232.43 | **0.002084** |
| Amphibian richness | -114.08 | -110.22 | 0.4615 |
| Total amphibian abundance | -198.01 | -193.42 | 0.3271 |
| Limnodynastes.dumerilii | -115.93 | -109.01 | **0.08591** |

| **TABLE S4:** Results from fixed effects contrasts for each supported global model. | | | | | |
| --- | --- | --- | --- | --- | --- |
| **Response metric** | **Model term** | **df** | **F ratio** | **Chisq** | **p value** |
| Reptile Richness | fire_status | 1 | 6.59 | 6.59 | **0.010** |
|  | baiting | 1 | 1.64 | 1.64 | 0.200 |
|  | rainfall | 1 | 0.22 | 0.22 | 0.638 |
|  | av_temp | 1 | 0.00 | 0.00 | 1.000 |
|  | fire_status:baiting | 1 | 0.33 | 0.33 | 0.565 |
|  | fire_status:rainfall | 1 | 5.22 | 5.22 | **0.022** |
|  | baiting:rainfall | 1 | 0.48 | 0.48 | 0.487 |
|  | fire_status:baiting:rainfall | 1 | 9.05 | 9.05 | **0.003** |
| *Anepischetosia maccoyi* | fire_status | 1 | 3.06 | 3.06 | **0.080** |
|  | baiting | 1 | 7.25 | 7.25 | **0.007** |
|  | rainfall | 1 | 2.37 | 2.37 | 0.124 |
|  | av_temp | 1 | 0.46 | 0.46 | 0.496 |
|  | fire_status:baiting | 1 | 1.26 | 1.26 | 0.261 |
|  | fire_status:rainfall | 1 | 0.11 | 0.11 | 0.738 |
|  | baiting:rainfall | 1 | 0.20 | 0.20 | 0.653 |
|  | fire_status:baiting:rainfall | 1 | 6.20 | 6.20 | **0.013** |
| *Pseudemoia entrecasteauxii* | fire_status | 1 | 1.94 | 1.94 | 0.164 |
|  | baiting | 1 | 0.96 | 0.96 | 0.327 |
|  | rainfall | 1 | 2.63 | 2.63 | 0.105 |
|  | av_temp | 1 | 2.19 | 2.19 | 0.139 |
|  | fire_status:baiting | 1 | 1.41 | 1.41 | 0.235 |
|  | fire_status:rainfall | 1 | 2.70 | 2.70 | 0.100 |
|  | baiting:rainfall | 1 | 10.50 | 10.50 | **0.001** |
|  | fire_status:baiting:rainfall | 1 | 0.95 | 0.95 | 0.329 |
| *Eulamprus tympanum* | fire_status | 1 | 4.69 | 4.69 | **0.030** |
|  | baiting | 1 | 7.01 | 7.01 | **0.008** |
|  | rainfall | 1 | 0.25 | 0.25 | 0.621 |
|  | av_temp | 1 | 0.78 | 0.78 | 0.377 |
|  | fire_status:baiting | 1 | 0.80 | 0.80 | 0.370 |
|  | fire_status:rainfall | 1 | 17.34 | 17.34 | **0.000** |
|  | baiting:rainfall | 1 | 0.44 | 0.44 | 0.509 |
|  | fire_status:baiting:rainfall | 1 | 13.20 | 13.20 | **0.000** |
| *Lampropholis guichenoti* | fire_status | 1 | 3.53 | 3.53 | **0.060** |
|  | baiting | 1 | 8.98 | 8.98 | **0.003** |
|  | rainfall | 1 | 0.54 | 0.54 | 0.464 |
|  | av_temp | 1 | 0.30 | 0.30 | 0.587 |
|  | fire_status:baiting | 1 | 0.83 | 0.83 | 0.364 |
|  | fire_status:rainfall | 1 | 0.67 | 0.67 | 0.415 |
|  | baiting:rainfall | 1 | 12.53 | 12.53 | **0.000** |
|  | fire_status:baiting:rainfall | 1 | 9.68 | 9.68 | **0.002** |
| *Limnodynastes dumerilii* | fire_status | 1 | 9.16 | 9.16 | **0.002** |
|  | baiting | 1 | 1.97 | 1.97 | 0.160 |
|  | rainfall | 1 | 0.01 | 0.01 | 0.940 |
|  | av_temp | 1 | 1.45 | 1.45 | 0.228 |
|  | fire_status:baiting | 1 | 0.17 | 0.17 | 0.685 |
|  | fire_status:rainfall | 1 | 1.95 | 1.95 | 0.163 |
|  | baiting:rainfall | 1 | 0.00 | 0.00 | 0.952 |
|  | fire_status:baiting:rainfall | 1 | 2.50 | 2.50 | 0.114 |

| **TABLE S5:** Pairwise comparisons for supported fixed effects contrasts for each supported global model. | | | | | | | | |
| --- | --- | --- | --- | --- | --- | --- | --- | --- |
| **Response metric** | **Baiting** | **Rainfall** | **Fire status** | **Contrast** | **ratio** | **SE** | **z ratio** | **p value** |
| Reptile Richness | Unbaited | Low | . | Unburnt / Burnt | 1.86 | 0.28 | 4.12 | **0.000** |
|  | Baited | Low | . | Unburnt / Burnt | 1.10 | 0.16 | 0.67 | 1.000 |
|  | Unbaited | Average | . | Unburnt / Burnt | 0.86 | 0.13 | -1.01 | 0.988 |
|  | Baited | Average | . | Unburnt / Burnt | 1.22 | 0.17 | 1.43 | 0.862 |
|  | . | Low | Unburnt | Unbaited / Baited | 1.13 | 0.18 | 0.73 | 0.999 |
|  | . | Low | Burnt | Unbaited / Baited | 0.67 | 0.09 | -3.15 | **0.019** |
|  | . | Average | Unburnt | Unbaited / Baited | 0.80 | 0.13 | -1.34 | 0.909 |
|  | . | Average | Burnt | Unbaited / Baited | 1.15 | 0.14 | 1.10 | 0.977 |
|  | Unbaited | . | Unburnt | Low / Average rainfall | 1.30 | 0.28 | 1.22 | 0.950 |
|  | Unbaited | . | Burnt | Low / Average rainfall | 0.60 | 0.12 | -2.63 | **0.098** |
|  | Baited | . | Unburnt | Low / Average rainfall | 0.93 | 0.20 | -0.36 | 1.000 |
|  | Baited | . | Burnt | Low / Average rainfall | 1.03 | 0.19 | 0.16 | 1.000 |
| *Anepischetosia maccoyi* | Unbaited | Low | . | Unburnt / Burnt | 6.12 | 5.17 | 2.14 | 0.325 |
|  | Baited | Low | . | Unburnt / Burnt | 0.61 | 0.37 | -0.81 | 0.998 |
|  | Unbaited | Average | . | Unburnt / Burnt | 1.01 | 0.62 | 0.02 | 1.000 |
|  | Baited | Average | . | Unburnt / Burnt | 2.42 | 0.94 | 2.27 | 0.246 |
|  | . | Low | Unburnt | Unbaited / Baited | 1.55 | 1.09 | 0.63 | 1.000 |
|  | . | Low | Burnt | Unbaited / Baited | 0.15 | 0.12 | -2.40 | 0.179 |
|  | . | Average | Unburnt | Unbaited / Baited | 0.24 | 0.13 | -2.56 | 0.119 |
|  | . | Average | Burnt | Unbaited / Baited | 0.57 | 0.26 | -1.22 | 0.951 |
|  | Unbaited | . | Unburnt | Low / Average rainfall | 1.27 | 0.99 | 0.31 | 1.000 |
|  | Unbaited | . | Burnt | Low / Average rainfall | 0.21 | 0.19 | -1.71 | 0.663 |
|  | Baited | . | Unburnt | Low / Average rainfall | 0.20 | 0.14 | -2.24 | 0.264 |
|  | Baited | . | Burnt | Low / Average rainfall | 0.77 | 0.45 | -0.44 | 1.000 |
|  | n/a | n/a | . | Unburnt / Burnt | 1.74 | 0.55 | 1.75 | **0.080** |
|  | n/a | n/a | . | Unbaited / Baited | 0.42 | 0.14 | -2.69 | **0.007** |
| *Pseudemoia entrecasteauxii* | . | Low | n/a | Unbaited / Baited | 0.22 | 0.12 | -2.87 | **0.016** |
|  | . | Average | n/a | Unbaited / Baited | 2.24 | 1.08 | 1.68 | 0.326 |
|  | Unbaited | . | n/a | Low / Average rainfall | 0.17 | 0.09 | -3.24 | **0.005** |
|  | Baited | . | n/a | Low / Average rainfall | 1.69 | 0.85 | 1.04 | 0.759 |
| *Eulamprus tympanum* | Unbaited | Low | . | Unburnt / Burnt | 7.36 | 2.93 | 5.01 | **0.000** |
|  | Baited | Low | . | Unburnt / Burnt | 1.37 | 0.44 | 0.98 | 0.992 |
|  | Unbaited | Average | . | Unburnt / Burnt | 0.42 | 0.17 | -2.17 | 0.303 |
|  | Baited | Average | . | Unburnt / Burnt | 1.15 | 0.39 | 0.42 | 1.000 |
|  | . | Low | Unburnt | Unbaited / Baited | 1.26 | 0.45 | 0.64 | 1.000 |
|  | . | Low | Burnt | Unbaited / Baited | 0.24 | 0.09 | -3.93 | **0.001** |
|  | . | Average | Unburnt | Unbaited / Baited | 0.42 | 0.18 | -1.98 | 0.442 |
|  | . | Average | Burnt | Unbaited / Baited | 1.15 | 0.34 | 0.49 | 1.000 |
|  | Unbaited | . | Unburnt | Low / Average rainfall | 4.73 | 2.90 | 2.53 | 0.130 |
|  | Unbaited | . | Burnt | Low / Average rainfall | 0.27 | 0.16 | -2.25 | 0.259 |
|  | Baited | . | Unburnt | Low / Average rainfall | 1.56 | 0.92 | 0.76 | 0.999 |
|  | Baited | . | Burnt | Low / Average rainfall | 1.32 | 0.70 | 0.52 | 1.000 |
| *Lampropholis guichenoti* | Unbaited | Low | . | Unburnt / Burnt | 0.75 | 0.27 | -0.79 | 0.999 |
|  | Baited | Low | . | Unburnt / Burnt | 3.99 | 1.77 | 3.11 | **0.022** |
|  | Unbaited | Average | . | Unburnt / Burnt | 1.96 | 0.84 | 1.57 | 0.773 |
|  | Baited | Average | . | Unburnt / Burnt | 0.79 | 0.32 | -0.58 | 1.000 |
|  | . | Low | Unburnt | Unbaited / Baited | 1.67 | 0.73 | 1.17 | 0.964 |
|  | . | Low | Burnt | Unbaited / Baited | 8.83 | 3.25 | 5.91 | **0.000** |
|  | . | Average | Unburnt | Unbaited / Baited | 1.41 | 0.66 | 0.73 | 0.999 |
|  | . | Average | Burnt | Unbaited / Baited | 0.57 | 0.21 | -1.55 | 0.786 |
|  | Unbaited | . | Unburnt | Low / Average rainfall | 1.80 | 1.07 | 0.98 | 0.991 |
|  | Unbaited | . | Burnt | Low / Average rainfall | 4.66 | 2.50 | 2.87 | **0.049** |
|  | Baited | . | Unburnt | Low / Average rainfall | 1.52 | 0.94 | 0.67 | 1.000 |
|  | Baited | . | Burnt | Low / Average rainfall | 0.30 | 0.17 | -2.15 | 0.322 |
| *Limnodynastes dumerilii* | n/a | n/a | . | Unburnt / Burnt | 2.73 | 0.91 | 3.03 | **0.002** |
